# Cross-cultural adaptation, validation, and arabic translation of the rhinoplasty outcome evaluation

**DOI:** 10.3389/fsurg.2026.1744891

**Published:** 2026-09-30

**Authors:** Feras Alkholaiwi, Yazeed Alghonaim

**Affiliations:** 1 Department of Otorhinolaryngology, Head and Neck Surgery, College of Medicine, Imam Mohammad Ibn Saud Islamic University (IMSIU), Riyadh, Saudi Arabia; 2 College of Medicine, Alfaisal University, Riyadh, Saudi Arabia

**Keywords:** adaptation, rhinoplasty, ROE questionnaire, translation, validation

## Abstract

**Background & aim:**

Rhinoplasty is among the most commonly performed cosmetic surgeries, yet patient satisfaction with this procedure tends to be lower compared to other facial aesthetic surgeries. To measure patient satisfaction following rhinoplasty, the Rhinoplasty Outcome Evaluation (ROE) questionnaire is employed. Therefore, this research was designed to determine the dependability and accuracy of the Arabic version of the ROE questionnaire.

**Methods:**

In this prospective study, the ROE questionnaire was administered to 76 patients who underwent rhinoplasty and seventy-four (74) volunteers who did not require functional or cosmetic nasal surgery. Started from June 2019 up to February 2021, The patients were followed up at 3 and 9 months after the procedure. To compare the initial distribution of questionnaire items between patients and control groups, an unpaired *t*-test was conducted. Internal consistency was assessed using Cronbach's *α* (Alpha). Hypothesis testing was performed with a significance threshold of 5%.

**Results:**

The control group exhibited a notably higher average total ROE score compared to the patient group, with scores of seventy-eight point six percent (78.6%) and forty-five point six per cent (45.6%), respectively (*P* < 0.001). Additionally, significant variations were identified in the mean scores of all questionnaire items when comparing baseline responses to both the first and second follow-up responses. The study tool's reliability, determined by Cronbach's *α* (Alpha), was reported to be 0.83.

**Conclusion:**

The Rhinoplasty Outcome Evaluation (ROE) questionnaire has been proven to be a reliable and valid tool for assessing outcomes in Arabic-speaking patients who have undergone rhinoplasty.

## Introduction

Rhinoplasty ranks among the most frequently performed cosmetic procedures by otorhinolaryngologists and plastic surgeons. This surgery is conducted not only for enhancing aesthetic appeal but also to improve nasal functionality. However, patient satisfaction is generally lower with rhinoplasty procedures than with other facial aesthetic surgeries (1, 2). Rhinoplasty is a complex and challenging procedure because it directly influences the patient's satisfaction with their own appearance, which in turn significantly impacts their self-esteem.

Most research on rhinoplasty has focused on aspects such as surgical techniques, surgeon expertise, complication development, postoperative results, and the frequency of reoperations. However, there is a notable lack of studies evaluating the procedure from the patient's viewpoint. Patients' motivations for undergoing rhinoplasty often differ from those of the surgeons performing the procedure. Some patients prioritize postoperative functionality, while others value both aesthetic improvements and functional outcomes. Satisfaction with rhinoplasty is influenced by various factors, including the social environment, educational background, personal experiences, and patient expectations. Therefore, assessing patient satisfaction is crucial and should be included in the criteria for determining the procedure's success.

There is a growing appeal in having patients self-report the outcomes of facial plastic surgery. Within this framework, the Quality of Life (QoL) questionnaire, which quantitatively evaluates the subjective results of surgical procedures, serves as an effective and appropriate tool for gauging patient satisfaction. This assessment is valuable for both clinicians and patients in determining the success and overall impact of the surgery. Quality of Life (QoL) is an individual's perception of their position in life, considering the cultural and value systems they are part of, and their personal goals, expectations, standards, and concerns. While general tools like the 36-Item Short Form Health Survey can measure QoL, a disease-specific questionnaire provides more precise insights. Specifically, the Rhinoplasty Outcome Evaluation (ROE) and the Functional Rhinoplasty Outcomes Inventory 17 (FROI-17) are designed to assess the outcomes of rhinoplasty.

The R.O.E. was developed by Alsarraf et al. in the year two thousand (2000) to specifically evaluate the outcomes of various aesthetic procedures, including rhinoplasty. Alsarraf et al. identified three key aspects of patient satisfaction in their questionnaire: physical, emotional, and social factors, all of which are associated with aesthetic procedures. The physical factor is associated with the patient's satisfaction with the nasal shape and its function, the emotional factor is associated with the patient's desire to change their appearance and the degree of confidence they have after the rhinoplasty procedure is completed, and the social factor involves the patient's family and their acceptance of the procedure (3).

The questionnaire consists of six questions (two from each of the key aspects). Since the ROE questionnaire has been used since 2001, it is considered the most appropriate questionnaire to assess a patient's QoL. This questionnaire has been translated and validated in multiple languages. For instance, Bulut et al. validated the German version of the ROE questionnaire, finding that the ROE-D is a reliable, validated, and sensitive tool for evaluating health-related Quality of Life (QoL) in rhinoplasty patients. In a similar vein, Izu et al. translated the ROE questionnaire into Brazilian Portuguese and confirmed its validity in assessing rhinoplasty outcomes. Additionally, Mulafikh et al. translated the questionnaire into Arabic, and their study showed that the Arabic version exhibited high reliability and validity.

Few studies have described rhinoplasty from the patient's point of view, which consists of assessing patient satisfaction and QoL, but to the best of our knowledge, there is only one study related to the Arabic population (4). Hence, there is an urgent requirement to assess the outcomes of the rhinoplasty procedure for this population using a valid tool that will help the surgeons and patients of Arabic-speaking countries. The objective was to ensure that the Arabic translation accurately measures patient satisfaction and outcomes following rhinoplasty, providing a dependable tool for clinical assessments in Arabic-speaking populations.

## Methods

### Study design

The Institutional Review Board and the Research Ethics Committee approved this study under Project Number 69-2019. All participating patients provided written informed consent. In this prospective study, which was started from June 2019 up to February 2021, the ROE questionnaire was administered at baseline to seventy-six (76) patients who subsequently underwent rhinoplasty. Follow-up questionnaires were administered to forty-seven (47) patients at two distinct timepoints: 3 months and 9 months following their rhinoplasty surgery. In addition, the questionnaire was completed by seventy-four (74) volunteers who accompanied the patients to the otorhinolaryngology clinic. These volunteers did not have any nasal symptoms and did not require either functional or cosmetic nasal surgery.

The main reasons of follow-up loss was included the impact of the COVID-19 pandemic, it was during the study period (June 2019 to February 2021), resulting in clinic closures and patients' reluctance to attend non-essential hospital visits. To assess potential attrition bias, baseline demographic characteristics and initial ROE scores were compared between patients who completed follow-up and those lost to follow-up; no statistically significant differences were observed between these groups (*P* > 0.05 for all comparisons).

To assess the reliability, particularly the inner consistency, of the ROE questionnaire, Cronbach's *α* (Alpha) was employed. A minimum threshold of zero point seven (0.7) was established as the acceptable standard for reliability. This statistical metric ensures that the questionnaire consistently measures the construct it is designed to evaluate.

The validity of the questionnaire, which assesses how well the instrument measures what it is supposed to measure, was determined using factor analysis. For this, Promax rotation was used for factor extraction with a maximum likelihood estimation method. This advanced statistical technique helps identify underlying relationships between measured variables. We hypothesized that a single factor would account for more than 50% of the variance among the questionnaire items, indicating a strong underlying construct being measured.

Furthermore, to determine the effect size of differences in mean values between the patient and volunteer groups at baseline, Cohen's *d* was utilized. This measure uses the pooled standard deviation to provide a standardized difference between means. Effect sizes were interpreted according to Cohen's guidelines (1988), which classify a small effect size as *d* = 0.2, a medium effect size as *d* = 0.5, and a large effect size as *d* = 0.8. These classifications help in understanding the magnitude of differences observed in the study.

Additionally, the proportion of variance explained by the questionnaire items was evaluated to understand how well the questionnaire items collectively account for the variability in patient responses. This evaluation provides insight into the overall explanatory power of the questionnaire, ensuring it is a robust tool for measuring outcomes in Arabic-speaking patients undergoing rhinoplasty.

### The questionnaire

The Arabic questionnaire translation comprises six items, maintaining the same structure as the original English ROE questionnaire. Each item is rated on a scale from zero to four (0–4), with higher scores reflecting more positive responses. The total score ranges from zero to twenty-four (0–24). For easier interpretation, the overall score is divided by twenty-four (24) and then multiplied by one hundred (100), resulting in a scale ranging from zero to one hundred percent (0%–100%). This conversion allows for a straightforward assessment of patient satisfaction levels. Two fluently bilingual otolaryngologists initially translated the English ROE into Arabic. Subsequently, the questionnaire was back-translated into English by two other qualified translators proficient in both languages. These back-translated versions were compared to the original ROE items, and the investigators reviewed and addressed any discrepancies.

### Statistical analysis

The statistical analyses were conducted using R software, version three point six point three (3.6.3). To summarize the distribution of categorical variables, frequencies and proportions were used, while means and standard deviations were applied to describe the distribution of questionnaire items. An independent samples *t*-test compared the baseline distribution of questionnaire items between patients and control subjects. Linear mixed modeling assessed whether changes in the total ROE score were statistically significant over time. For *post-hoc* analysis, paired *t*-tests with Tukey adjustments were performed to make pairwise comparisons. Hypothesis testing was conducted with a significance threshold set at five per cent (5%).

## Results

### Descriptive statistics

At baseline, seventy-six (76) patients and seventy-four (74) control subjects completed the questionnaire. The demographic details of participants in each group are detailed in Table 1. The control group had a significantly higher average age compared to the patient group (*P* < 0.001). Additionally, the patient group had a notably higher proportion of females [ninety-eight point seven percent (98.7%)] compared to the control group [forty-seven point three percent (47.3%)], with this difference being statistically significant (*P* < 0.001). However, there was no significant difference in the distribution of nationalities between the two groups. On average, patients had undergone nasal surgery four point fourteen (4.14) months prior, with a standard deviation of one point sixty-seven (1.67) months.

**Table 1:** Demographic characteristics of the study sample groups.

Demographic details	Patients	Controls	*P* value
	*N* = 76	*N* = 74	
Age	28.4 (5.78)	35.0 (9.02)	<0.001
Gender:			<0.001
Female	75 (98.7%)	35 (47.3%)	
Male	1 (1.32%)	39 (52.7%)	
Nationality:			0.747
Saudi	72 (94.7%)	71 (95.9%)	
Iraqi	1 (1.32%)	0 (0.00%)	
Jordanian	1 (1.32%)	1 (1.35%)	
Kuwaiti	0 (0.00%)	1 (1.35%)	
Yemeni	2 (2.63%)	0 (0.00%)	
Egyptian	0 (0.00%)	1 (1.35%)	

The data are displayed as counts and percentages for categorical variables, providing a clear view of the distribution within different categories. For continuous variables, the data are represented as the mean value along with the standard deviation (SD), offering insight into the average values and the variability within the dataset.

### ROE scores for the patients and controls

Table 2 shows the differences in the mean responses between the groups for the individual items, as well as the total scores. We found that the average total ROE score was significantly higher for the controls than it was for the patients (78.6 vs. 45.6%, *P* < 0.001). The average score for each questionnaire item was higher for the control group compared to the patient group, except for Item 2, which asks, “How well are you able to breathe through your nose?” Cohen's test was employed to calculate the effect size using the standardized mean difference. The results indicate that there was a large effect size for all the items except for Item 2, suggesting that the questionnaire has good discriminating power. Figure 1 shows the responses to the individual items. The results show better responses for the control group than for the patients, as shown by the higher proportions of participants who responded positively.

**Table 2:** Comparison of the individual and total baseline ROE scores between the groups.

ROE Questions	Patients	Controls	*P* value	Cohen's *d*
	*N* = 76	*N* = 74		
How satisfied are you with the appearance of your nose?	1.31 (1.00)	2.81 (1.02)	0.001	−1.4
How well can you breathe through your nose?	2.84 (1.13)	3.16 (0.94)	0.060	−0.31
How do you feel your friends and loved ones like the appearance of your nose?	1.75 (1.08)	2.82 (1.06)	<0.001	−1
Do you feel that your nasal appearance limits your social or professional activities?	2.71 (1.21)	3.84 (0.50)	0.001	−1.21
How confident are you that your nasal appearance is optimal?	1.20 (1.01)	2.96 (1.10)	0.001	−1.66
Would you consider surgically altering the appearance or function of your nose?	1.12 (1.06)	3.27 (0.93)	0.001	−2.15
Total score	45.6 (17.3)	78.6 (15.5)	0.001	−2

The information is displayed as counts and percentages for categorical variables, while continuous variables are represented by the average (mean) along with the standard deviation (SD). Statistical comparisons were made using an unpaired *t*-test. Additionally, seventy-four (74) participants were included in the analysis, ensuring robust data interpretation. This methodology allows for a clear differentiation between types of variables and facilitates precise statistical evaluation.

**Figure 1:**
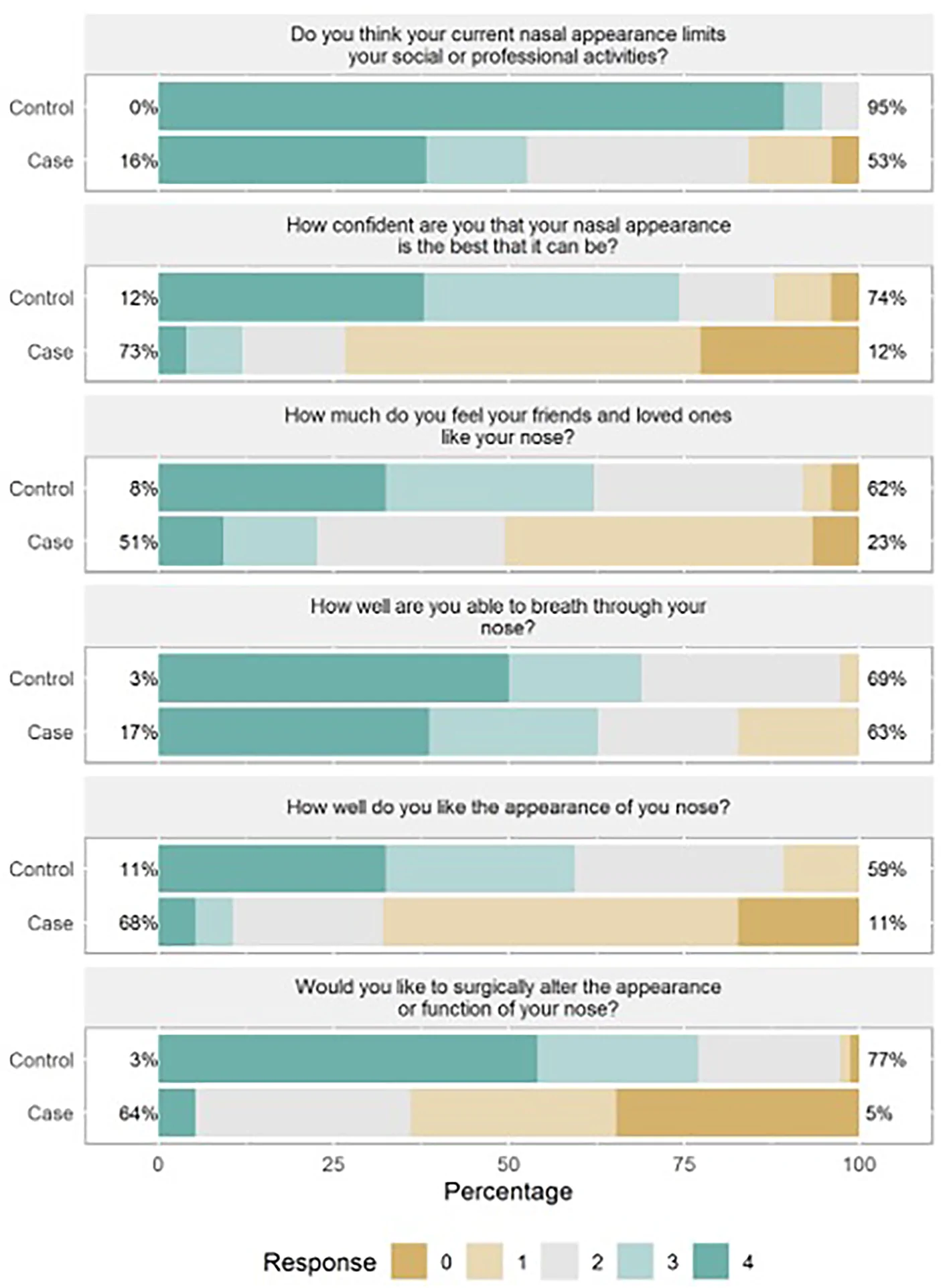
Baseline responses to the individual questionnaire items.

### Changes in the ROE scores for the patients

The linear mixed modeling analysis revealed a change in the total ROE score [*F*(2, 118) = 68.48, *P* < 0.001]. The results of the pairwise comparisons are presented in Table 3. Detailed results of the pairwise comparisons are displayed in Table three (3). Significant differences were found in the mean response scores for all items, except Item two (2), between the baseline and the first follow-up, as well as between the baseline and the second follow-up. Notably, the mean score for Item two (2) remained consistent across all timepoints. There were no statistically significant differences between the follow-up timepoints (*P* > 0.05, for all comparisons).

**Table 3:** Changes in the individual and total ROE item scores.

ROE items questions	Baseline (B)	Time 1 (T1)	Time 2 (T2)	*P* overall	*P* B vs. T1	*P* B vs. T2	*P* T1 vs. T2
How well do you like the appearance of your nose?	1.39 (1.08)	2.57 (0.99)	2.40 (0.99)	0.001	<0.001	<0.001	0.700
How effectively can you breathe through your nose?	2.85 (1.11)	3.13 (0.95)	3.11 (0.96)	0.335	0.378	0.435	0.994
To what extent do you believe your friends and family appreciate the appearance of your nose?	1.91 (1.15)	2.70 (1.06)	2.64 (1.09)	0.001	0.002	0.005	0.957
Do you feel that the current look of your nose restricts your social or work-related activities?	2.62 (1.23)	3.47 (0.86)	3.49 (0.91)	0.001	<0.001	<0.001	0.994
How confident are you that your nose looks as good as it possibly can?	1.28 (1.09)	2.72 (1.08)	2.64 (0.94)	0.001	<0.001	<0.001	0.917
Are you interested in undergoing surgery to change the appearance or improve the function of your nose?	1.09 (1.15)	2.68 (1.32)	2.87 (1.08)	0.001	<0.001	<0.001	0.715
Total score	46.5 (19.4)	72.0 (19.8)	71.5 (17.2)	0.001	<0.001	<0.001	0.990

The information is conveyed as counts and percentages for categorical variables, while continuous variables are represented by the average (mean) along with the standard deviation (SD). Statistical evaluations were conducted using a paired *t*-test. Furthermore, *P*-values for the *post-hoc* pairwise comparisons were adjusted employing the Tukey correction. In total, seventy-four (74) participants' data were analyzed, ensuring comprehensive statistical interpretation.

### Validation of the ROE questionnaire

The baseline data from the patients and controls (*n* = 150) were used to validate the ROE questionnaire (Table 4). The results show that one factor explained 52.35% of the variance among all the ROE questionnaire items. When Item two (2) was excluded from the analysis, the percentage of variance explained by a single factor increased to sixty-two percent (62%). In both scenarios, the percentage of variance explained by a single factor was deemed satisfactory. Factor loadings exceeded zero point seven (0.7) for all items except Item two (2). The reliability of the questionnaire, measured by Cronbach's *α* (Alpha), was zero point eighty-three (0.83), which is considered adequate.

**Table 4:** Factor analysis results.

ROE Questions	Loading
How satisfied are you with the way your nose looks?	0.88
How effectively can you breathe through your nose?	0.18
To what extent do you believe your friends and family appreciate the appearance of your nose?	0.71
Do you feel that the current look of your nose restricts your social or professional activities?	0.66
How confident are you that your nose looks its best?	0.9
Are you interested in undergoing surgery to change the appearance or improve the function of your nose?	0.76

### Attrition analysis

Comparison of baseline characteristics between patients who completed follow-up (*n* = 47) and those lost to follow-up (*n* = 29) revealed no significant differences in age (28.1 ± 5.6 vs. 28.9 ± 6.1 years, *P* = 0.54), gender distribution (*P* = 0.89), or baseline total ROE scores (44.9 ± 17.1 vs. 46.7 ± 17.8%, *P* = 0.65). These findings suggest that attrition was likely random and did not introduce systematic bias into the longitudinal analysis.

## Discussion

Rhinoplasty is regarded as one of the most complex plastic surgeries to perform. Despite its intricate nature and the challenges involved, it remains one of the most sought-after aesthetic surgeries worldwide, consistently ranking among the top five cosmetic procedures. The International Society of Aesthetic Plastic Surgery (ISAPS) Global Survey on Aesthetic/Cosmetic Procedures for two thousand nineteen (2019) reported a seven point four percent (7.4%) increase in cosmetic procedures in two thousand nineteen (2019) compared to the previous year, with rhinoplasty ranking fifth among surgical procedures performed by plastic surgeons. However, due to the inherent complexities of the surgery, rhinoplasty also has one of the lowest patient satisfaction ratings.

Patient satisfaction is vital for any cosmetic surgical procedure, as it not only affects the patient's quality of life but also the outcome of the surgical procedure. Therefore, it is very important to assess patient satisfaction, not only for the treatment of the patient but also for research purposes. Patient satisfaction can be influenced by various factors, including social, environmental, and educational factors, as well as life experience and the patient's expectations.

Several tools have been created to assess postoperative results and patient satisfaction. One such tool is the Rhinoplasty Outcome Evaluation (ROE) questionnaire, developed by Alsarraf in the year two thousand (2000). Alsarraf aimed to ensure that the questionnaire was straightforward and easy to administer, addressing concerns about its simplicity during its development. However, after many years of use, the questionnaire is still very popular worldwide due to its simple, short, and easy-to-understand format (3). Indeed, the questionnaire has been used, applied, and translated multiple times (5–7).

We found that our study population is younger than those reported in previous studies, with the original questionnaire study reporting an average age of thirty-four (34) years and the Spiekermann et al. study reporting an average age of thirty point one (30.1) years. Moreover, a statistically significant difference in sex distribution was observed between patients and control subjects (*P* < 0.001). This result aligns with the findings of Çelik et al., who also reported significant differences in age and sex distributions between their study patients and control subjects, with *p*-values of (0.108) and (0.110), respectively. In contrast, Izu et al. did not identify significant differences between their groups. Similarly, Mulafikh et al. noted no significant age differences between patients and controls, although they did report a significant difference in sex distribution between the two groups.

We found that the questionnaire has a high validity, as it can discriminate between healthy controls and rhinoplasty candidates. The average total ROE score in our study was significantly higher for the controls than it was for the patients (78.6 vs. 45.6%, *P* < 0.001), which is in line with the findings of Izu et al., who observed that the scores for the control subjects were higher (17.94) than those of the patients (6.6) (7). Çelik and colleagues observed that participants in the study group had significantly higher scores on questions E1-E5 and overall T-OAR scores than those in the control group (*P* < 0.05). Similarly, Bulut and co-researchers validated the ROE-T's effectiveness in distinguishing patients requiring rhinoplasty from the general population, with scores of 3.3 ± 2.3 for the general population vs. 15.8 ± 5.5 for rhinoplasty patients (*P* < 0.001). Spiekermann and his team reported that patients had notably higher average scores on the German version of the Utrecht Questionnaire for Outcome Assessment in Aesthetic Rhinoplasty (D-OAR) compared to healthy individuals (14.9 ± 5.3 vs. 5.3 ± 0.8, *P* < 0.001). In a similar vein, Mulafikh DS and colleagues identified significant differences in both individual question scores and total scores between rhinoplasty patients and control subjects, with *P*-values ranging from 0.005 to 0.0001. These findings, along with our research, confirm that the ROE questionnaires possess adequate discriminant validity, enabling them to effectively differentiate between rhinoplasty patients and healthy controls.

The primary limitation of the ROE questionnaire is that it places more emphasis on the cosmetic aspects than the functional aspects of rhinoplasty. Out of the six questions, five are focused on cosmetic outcomes, while only one question (“How well are you able to breathe through your nose?”) addresses the functional aspect of patient satisfaction. To address this limitation and give more weight to functional outcomes, the Functional Rhinoplasty Outcome Inventory 17 (FROI-17) was developed, which is slightly longer than the ROE questionnaire. In our study, the control group had higher average scores for all items except for Item 2, which asks, “How well are you able to breathe through your nose?” The effect size was calculated using Cohen's *d* and the standardized mean difference. The results indicate that there was a large effect size for all the items except Item 2, suggesting that the questionnaire has good discriminating power. In addition, the factor analysis shows that the loadings were greater than 0.7, except for Item 2. This item explained 52.35% of the variance among all the ROE items. When Item 2 was excluded, the % variance explained by a single factor increased to 62%. Mulafikh et al. also found a low score for Item 2 (4). These results indicate that the questionnaire addresses more aesthetic concerns than functional.

The internal consistency of the questionnaire, assessed through Cronbach's *α* (Alpha) [with a minimum acceptable value set at zero point seven (0.7)], indicates the homogeneity of the ROE questionnaire items. Our study found a Cronbach's *α* (Alpha) of zero point eighty-three (0.83), which is consistent with the results of Alsarraf et al., who reported a Cronbach's *α* (Alpha) of zero point eighty-three (0.83) six months postoperatively and zero point eighty-eight (0.88) one year after surgery. Spiekermann et al. documented a Cronbach's *α* (Alpha) of zero point ninety-one (0.91) before surgery, and values of zero point ninety-two (0.92) at one month, zero point ninety-two (0.92) at three months, and zero point ninety-one (0.91) at twelve months post-surgery, demonstrating excellent internal consistency at all evaluated time points. Çelik et al. also observed high internal consistency for the ROE-T questionnaire, with Cronbach's *α* (Alpha) values of zero point eight eight seven (0.887) preoperatively and zero point seven nine eight (0.798) postoperatively. Another study by Çelik and Demirbilek reported that the T-OAR questionnaire exhibited high internal consistency, with a Cronbach's *α* (Alpha) of zero point eight (0.8). Mulafikh et al. found good internal consistency for the ROE-A, with a Cronbach's *α* (Alpha) of zero point seventy-eight (0.78). Additionally, Rosa et al. reported Cronbach's *α* (Alpha) values of zero point eighty-eight (0.88) for preoperative responses and zero point eighty-six (0.86) for postoperative responses.

Several studies have employed the ROE questionnaire and have documented significant impacts of rhinoplasty on patients' quality of life. This underscores the importance of follow-up to evaluate long-term satisfaction among these patients.

The duration of follow-up has varied significantly across different studies. In our research, we assessed the test-retest reliability of the ROE questionnaire by comparing scores at the initial assessment and at two subsequent follow-up points. There were statistically significant differences in the mean scores for all items between the baseline and the first follow-up, as well as between the baseline and the second follow-up. However, no significant differences were found between the two follow-up points (*P* > 0.05).

In a study by Arima et al., follow-up periods ranged from six (6) months to ten (10) years, and they found no significant statistical changes in quality of life associated with the length of the follow-up period. Conversely, Alsarraf et al. reported a notable improvement in ROE-T scores three (3) months post-surgery (3.3 ± 2.3 vs. 22.2 ± 2.8, *p* < 0.001) (8). These findings provide evidence that the ROE is a highly responsive questionnaire that affects rhinoplasty outcomes and, therefore, the patient's QoL. Izu et al. investigated the reason for the differences in the ROE scores at different follow-up timepoints after surgery. They followed patients for 15 days and then until the third postoperative month, and they found that the difference in the ROE scores was attributed to the decrease in postoperative edema at the later timepoint (5, 7). Bulut showed good results and acceptable internal consistency at 6 months and 1 year after surgery (6). In a retrospective cohort study, Helling et al. showed a significant improvement in the ROE scores for 90 patients after rhinoplasty revision (9). Although the ROE questionnaire outcomes did not change significantly over longer follow-up periods or between the two follow-up periods, we found significant changes in the individual and total ROE item scores between the baseline and the first follow-up. Hence, we suggest that the ROE questionnaire is sensitive enough to detect changes in patient satisfaction soon after the operative procedure and at the first follow-up.

A few limitations were present in our study related to the small sample size and the shorter follow-up period. While Modern Standard Arabic is shared, dialectal/cultural differences and sampling from a specific setting may limit generalizability across all Arabic-speaking populations. Further validation across different Arabic-speaking countries/dialects would be valuable. In addition, in this study no comparison of the preoperative ROE scores with the postoperative scores.

## Conclusion

The ROE questionnaire has been confirmed as a valid and reliable tool for assessing outcomes in Arabic-speaking rhinoplasty patients. Our study's findings align with those of previous research in other languages suggesting its potential applicability across different population. Although our sample size is considered small, demographic imbalance between groups, along with limited psychometric analysis. Therefore, it is recommended that future studies with larger and more divers samples, on similar topics incorporate the ROE questionnaire to ensure consistency and accuracy in measuring patient satisfaction and surgical success. Expanding its use in diverse populations will enhance its robustness and provide a broader understanding of rhinoplasty outcomes across different demographic groups.

## Data Availability

The original contributions presented in the study are included in the article/Supplementary Material, further inquiries can be directed to the corresponding author.
