# Supplementary material for: Cross-cultural adaptation, validation, and arabic translation of the rhinoplasty outcome evaluation
Source: Front Surg. 2026 Sep 30;13:1744891. doi: 10.3389/fsurg.2026.1744891 (PMC13661970; doi:10.3389/fsurg.2026.1744891)
Supplement: Supplementary file 1 [file Supplementaryfile1.docx]

**Evaluation of the results of rhinoplasty**

**This questionnaire is designed to help the surgeon determine the best outcomes for the client after rhinoplasty. We treat your feedback with strict confidentiality, but may be used to improve the outcomes of future surgical procedures for clients. *(Quality of Life Measurement Tool)**

**Please select the number that reflects your actual opinion of the following questions:**

| **1. How satisfied are you with the shape of your nose?** | | | | |
| --- | --- | --- | --- | --- |
| **Never satisfied**  **0** | **Somewhat satisfied**  **1** | **Acceptable**  **2** | **very satisfied**  **3** | **Completely satisfied**  **4** |
| **2. How well can you breathe through your nose?** | | | | |
| **I can't never.**  **0** | **I can to some extent**  **1** | **Acceptable**  **2** | **very big**  **3** | **I can totally**  **4** |
| **3. How much do you feel that your friends and loved ones like the look of your nose?** | | | | |
| **Completely dissatisfied**  **0** | **To some extent**  **1** | **Acceptable**  **2** | **Too much**  **3** | **Completely satisfied**  **4** |
| **4. Do you think the current appearance of your nose limits your social and professional activities?** | | | | |
| **Always**  **0** | **Probably**  **1** | **Sometimes**  **2** | **rarely**  **3** | **Never**  **4** |
| **5. How confident are you that your current nose looks the best it can be?** | | | | |
| **Completely dissatisfied**  **0** | **To some extent**  **1** | **Acceptable**  **2** | **very satisfied**  **3** | **Completely satisfied**  **4** |
| **6. Do you want to change the appearance or function of your nose through surgery?** | | | | |
| **Sure.**  **0** | **Most likely yes**  **1** | **possibly**  **2** | **Probably not.**  **3** | **no**  **4** |

Dr. Firas Al-Khaliwi, Dr. Yazid Al-Ghoneim
